# Oxygen saturation trends in the first hour of life in healthy full-term neonates born at moderate altitude

**DOI:** 10.12669/pjms.294.3848

**Published:** 2013

**Authors:** Hamed Said Habib

**Affiliations:** Dr.amed Said HabibDean, Rabigh College of Medicine, Associate Professor and Consultant of Pediatrics, Pediatric Department, Faculty of Medicine, King Abdulaziz University, Saudi Arabia.

**Keywords:** Pulse oximetry, Cardiopulmonary transition, Moderate altitude, Oxygen saturation

## Abstract

***Background: ***Transition from a parallel circulation in utero to an in-series circulation immediately after birth is partly an oxygen-dependent process. Relative hypoxemia with increasing altitude above sea level exerts a certain degree of stress on oxygen-dependent metabolic processes throughout the body.

***Objective: ***The present study aimed to determine the reference values for oxygen saturation and the pre-ductal and post-ductal oxygen saturation trends during the first 60 min of life in healthy full-term neonates born at moderate altitude (1500-2500 m) using pulse oximetry.

***Methods:*** This descriptive study was carried out over a period of three months started from July 2011 in the Neonatology Department of King Abdulaziz Specialist Hospital, Taif, Saudi Arabia. In this observational study, arterial oxygen saturation in the right hand and right foot of each infant was recorded by pulse oximetry immediately after birth and continuously within the first 60 min of life. The respiratory rate, heart rate, and blood pressure were measured at birth and at 1 h after birth. Cord blood gas and haemoglobin levels were also measured.

***Results:*** The study was conducted in a hospital situated at an altitude of 1640 m above sea level. Immediately after birth, the mean pre-ductal SpO_2_ in the right hand was 68% (51–80%); in the right foot, the mean post-ductal SpO_2_ was 60% (40–77%). This difference was statistically significant (p < 0.01); however, it became statistically insignificant at 20 min (4–45 min) and disappeared at 25 min, when the SpO_2_ in both limbs equalised at 88% (83–96%). SpO_2_ levels > 94% were reached after 13 min (4–35) min pre-ductally and after 22 min (10–45 min) post-ductally. The mean respiratory rate, heart rate, and mean blood pressure at birth were 56/min, 140/min, and 34 mmHg, respectively; at 60 min, they were 40/min, 123/min, and 47 mmHg, respectively.

***Conclusion:*** This study defined normal range of SpO_2_ values in healthy full-term neonates born at moderate altitude in the first 60 minutes of life. These are expected to serve as base line data for normal neonates born at similar altitudes. With regard to pre-ductal and post-ductal oxygen saturation levels, cut-off values lower than those used at sea level should be adopted for neonates born at moderate altitudes.

## INTRODUCTION

With increasing altitudes above sea level, the oxygen-carrying capacity of haemoglobin progressively reduces. This occurs because the partial pressure of oxygen in ambient air and in air inspired into the lung decreases; therefore, the amount of oxygen available for diffusion into the bloodstream also decreases. The resulting relative hypoxemia stresses oxygen-dependent metabolic processes throughout the body. Early in the process of cardiopulmonary transition, immediately after birth, and before the closure of the ductus arteriosus, the arterial oxygen saturation (SpO_2_) in the ascending aorta is higher than that in the descending aorta.Many studies have documented a difference in oxygen saturation between the upper extremities (pre-ductal) and lower extremities (post-ductal), with lower oxygen saturation observed in post-ductal sites within the first hour of life. Placing twin oximeter probes on the right hand and either foot appears to be an effective method of identifying right to left ductal shunting.

The transition from the parallel circulation in utero to the in-series circulation after birth results in a higher overall arterial oxygen content. The time required for this increase in oxygenation is partially dependent on the presence of residual cardiopulmonary shunts and available oxygen. Since the closure of the ductus arteriosus is an oxygen-dependent process, it remains unclear whether the relative hypoxaemia that occurs at moderate altitudes would affect the transition time and the oxygen saturation trends within the first 60 minutes of life. Limited studies have evaluated the transition period in situations where the availability of environmental oxygen is low, similar to that in moderate altitudes. Interestingly, in 2005, Bakr and Habib reported that the normal values of SpO_2_ as measured by pulse oximetry and other vital parameters in full-term neonates born at an altitude of 1640 m differed from corresponding data in similar neonates born at sea level. Awareness of the risk factors, the clinical signs of hypoxemia in newborn infants, and SpO_2_ measurements by pulse oximetry can aid health professionals and parents in recognising and preventing altitude-associated cardiac and pulmonary illness.

The present study primarily aimed to determine trends and reference values for pre-ductal and post-ductal oxygen saturation by pulse oximetry during the first 60 min of life in healthy full-term infants born at moderate altitude. The establishment of appropriate cut-off values will aid neonatologists in the accurate detection and optimal management of cardiac and pulmonary disorders affecting neonates born at moderate altitudes.

## METHODS

This descriptive study was carried out in the Neonatology Department of King Abdulaziz Specialist Hospital (KAASH), Taif, Saudi Arabia, which is located at an altitude of 1640 m above sea level from 1478 to 1897 m. The study was approved by the ethical committee of the hospital. Parental written informed consent was obtained prior to the inclusion of infants in the study. KAASH is the main maternity hospital in Saudi Arabia and has the largest newborn unit in the area (an average of 12000 deliveries/year). The inclusion criteria for this study were as follows: gestational age > 36 weeks; birth weight, 2,500–4,200 g; and healthy infants with stable clinical condition without any evidence of hypoxia, history of maternal illness, obstetric complications, or congenital anomalies.

The study included the first 100 infants delivered in KAASH during the working hours of the investigators who fulfilled the inclusion criteria. Routine post-natal care of the newborns with normal Apgar scores took place independent of the research team. Immediately after birth, all infants underwent oropharyngeal and nasal suctioning and were placed under a warmer. There was no indication for oxygen administration in any of the infants. Within the first minutes of life, the infant’s right hand and right foot were cleaned and pulse oximeters (Digioxi PO920; Digicare Biomedical Technology) were attached to the palm of the right hand and on to the big toe of the right foot. Limbs were held steady to avoid motion, and interference from heat and light were prevented by shielding. To avoid the effect of limb movement, the pulse waveform was observed until there was a strong steady signal with a pulse rate matching that on the cardiac monitor. Data reflecting SpO_2_ from the right upper and lower limbs (RUL and RLL, respectively) were collected at 1, 3, 5, 10, 15, 20, 25, 30, 45 and 60 min after birth; further, the time point at which SpO_2_ equality was achieved between the upper and lower limbs was also recorded. Other data included the respiratory rate, heart rate, and blood pressure at birth and 1 hour of life. Cord blood gas and haemoglobin levels were also measured.

The collected data were entered in a database and statistical analysis was performed. A confidence interval of 95% was defined, and results were analysed with Student’s *t*-tests for parametric data. P < 0.05 was considered statistically significant.

## RESULTS

The present study lasted for 3 months and eventually included 100 full-term healthy newborn babies. Apgar scores ranged from 8 to 10 at one and five minutes both. Neonates who required and received oxygen supplementation at the time of birth were excluded from analysis. A summary of patient characteristics is presented in Table-I. Pre-ductal and post-ductal SpO_2_ values were obtained from all 100 neonates at 1, 3, 5, 10, 15, 20, 25, 30, 45 and 60 min after birth. The mean values for pre-ductal and post-ductal SpO_2_ levels, along with the ranges, are shown in Table-II.

At the first minute of life, the mean pre-ductal SpO_2_ (RUL) was 68% (range, 51–80%) and the mean post-ductal SpO_2_ (RLL) was 60% (range, 40–77%). This difference was statistically significant (p < 0.01); however, it became statistically insignificant at 20 minutes and disappeared completely at 25 min (range, 4–45 min) at which point the SpO_2_ levels in both the upper and lower limbs were equalised (mean, 88%; range, 83–96%). SpO_2_ > 94% was achieved pre-ductally at 13 min after birth (range, 4–35 minutes) and post-ductally at 22 minutes after birth (range, 10–45 minutes). The mean respiratory rate, heart rate, and mean blood pressure at birth were 56/ minutes, 140/ minutes, and 34 mmHg, respectively; at 60 minutes, these were 40/ minutes, 123/ minutes, and 47 mmHg, respectively. A summary of data for vital parameters at birth and at one hour of life along with the cord blood pH and haemoglobin levels are demonstrated in Table-III.

## DISCUSSION

Within seconds after birth, the lungs fill with air, the pulmonary blood flow increases, and the pulmonary blood pressure drops, as foetal shunts through the foramen ovale and ductus arteriosus are closed. The availability of oxygen profoundly influences the nature and intensity of the developmental cardiopulmonary changes that occur in the peri-natal period which involves the transformation of the foetus to a newborn infant. At high altitudes, such changes differ clearly from those occurring at sea level, with differences in oxygen arterial saturation, breathing patterns, maturation of respiratory control reflexes, and velocity of regression of the foetal characteristics of the pulmonary vasculature. Despite the limitations of pulse oximetry, it has become a vital instrument in the medical care of infants and children, particularly for monitoring infants in the delivery room. Continuous monitoring by pulse oximetry is now a part of routine clinical care for nearly all patients requiring oxygen therapy, particularly in neonatal units. 

Pulse oximetry in general is a safe, feasible test that adds value to existing screening techniques for congenital heart disease. It can detect cases of critical congenital heart disease that go undetected with antenatal ultrasonography; further, the early detection of other diseases is an additional advantage. Post-ductal arterial pulse oximetry screening during the first 24 hours of life is currently considered the most useful strategy to prevent circulatory collapse or death associated with congenital heart disease. Therefore, altitude-adjusted cut-off levels for pre-ductal and post-ductal SpO_2_ values are needed for accurate interventional decisions. However, few studies have measured pulse oxygen in neonates and infants born at moderate and high altitudes. A wide disparity has been noticed during attempts to define normal values of oxygen saturation in paediatric age groups at varying altitudes. In a study conducted at 1640 m above sea level by the author of the present study, the mean SpO_2_ value at 1 hour and 24 hours after birth was 94.3 and 95.4%, respectively. The SpO_2_ values obtained by pulse oximetry have been reported to vary with the level of the infant’s activity and with altitude. Children living at high altitudes have lower oxygen saturation than children at sea level, a phenomenon that manifests only above the altitude of 1600 m.

Several studies using pulse oximetry in the delivery room conducted at sea level have documented that it requires longer than 5–10 min for a newborn undergoing normal post-natal transition to attain oxygen saturation near 80%. Both pre-ductal and post-ductal SpO_2_ levels rise gradually and generally do not reach 90% in the first 5 min of life. To the best of the author’s knowledge, the present study is the first to evaluate normal cut-off levels for SpO_2_ values at the moment of cardiopulmonary transition in healthy full-term neonates born at an altitude greater than 1600 m.

The present findings demonstrated that in healthy neonates born at an altitude of 1640 m, there exists a significant difference between the pre-ductal and post-ductal SpO_2_ levels during the first 20 minutes of life. These data indicate that neonates remain relatively desaturated at moderate altitudes, delaying the time needed for functional closure of the ductus arteriosus, with SpO_2_ levels increasing steadily towards the normal range during the first 20 minutes of life. Both pre-ductal and post-ductal SpO_2_ levels did not reach 90% in the first 25 minutes of life at moderate altitude in the present study. These findings could be explained by the presence of right-to-left shunting at the ductus arteriosus, leading to lower SpO_2_ levels in the lower limbs due to the flow of venous blood into the descending aorta.

**Table-I:** A summary of patient characteristics

	*Gestational age*	*Birth weight*	*Apgar score*	
	*Months*	*Kg*	*1 min*	*5 min*
Min	38	2.6	8	9
Max	41	4.1	9	10
Mean	39.4	3.12	9	9

**Table-II:** Pre-ductal and post-ductal SpO_2_ values through the first hour after birth

	*MIN 1*	*MIN 3*	*MIN 5*	*MIN 10*	*MIN 15*	*MIN 20*	*MIN 25*	*MIN 30*	*MIN 45*	*MIN 60*
RUL SpO_2_ pre-ductal	68 * (51–80)	70* (56–85)	73* (68–94)	78* (71–96)	82* (74–97)	85 (76–97)	88 (84–98)	91 (88–98)	93 (90–98)	94 (91–99)
RLL SpO_2_ post-ductal	60* (40–77)	63* (44–81)	66* (53–85)	71* (61–94)	75* (67–95)	79 (72–96)	88 (83–96)	90 (85–98)	91 (90–97)	94 (90–99)

Data are presented as mean (range). *P < 0.05 for differences between pre-ductal and post-ductal SpO_2_ levels

**Table-III:** Vital signs and cord blood characteristics

	*Heart Rate*		*Respiratory Rate*		*Mean Blood Pressure*		*Cord Blood*	
	*Birth*	*60 Min*	*Birth*	*60 Min*	*Birth*	*60 Min*	*pH*	*Hb (g/dL)*
Min	140	123	56	40	31	34	7.26	12.7
Max	200	168	80	62	57	63	7.37	19.4
Mean	169	150	65	55	42	47	7.31	15.85
SD	15.39	10.63	5.54	5.08	7.11	7.84	0.04	1.88

In a study conducted at Buenos Aires, Argentina, the pre-ductal and post-ductal arterial oxygen saturation levels were measured in 110 healthy newborn infants immediately after birth at sea level, with mean SpO_2_ values of 89% and 81%, respectively.^12^ They also demonstrated that in healthy newborn infants, a significant difference existed between the pre-ductal and post-ductal SpO_2_ levels during the first 15 min of life. Further, a study performed at the University of Munich showed statistically significant differences between the pre-ductal and post-ductal SpO_2_ values at two and five minutes after birth; moreover, the magnitude of this difference decreased after 10 minutes and the threshold of 95% SpO_2_ was achieved pre-ductally at 12 minutes (range, 2–55 minutes) and post-ductally at 14 minutes (range, 3–55 minutes).^13^

Another study at Mount Sinai Medical School, New York, reported that during the first 10 minutes of life in 90% of the neonates examined, the SpO_2_ recorded over the right ulnar region (pre-ductal) was higher than that obtained at the Achilles tendon (post-ductal).^14^ Further, the SpO_2_ recorded on the right hand at 10 minutes was 91.2 ± 3.2 while that on the right leg was 87.1 ± 5.7, indicating that at 10 minutes, these differences diminished and almost completely disappeared.^14^

There are two main limitations in this study that are important to note. First, there were a relative small number of subjects and this limited our statistical power. Second, there was no control group done at sea level by the same protocol. Therefore, this study is providing a base line data rather than reference values.

While the normal pre-ductal and post-ductal SpO_2_ values at sea level within the first hour after birth are well known^11^, the present study is the first to define normal pre-ductal and post-ductal SpO_2_ values at moderate altitude within the first hour of life.

## CONCLUSION

The present findings indicated that the time for the transition from the parallel circulation in utero to the in-series circulation after birth was significantly lower at the moderately high altitude of 1640 m. This emphasises the importance of adopting lower pre-ductal and post-ductal oxygen saturation cut-off levels than those used at sea level for determining the need for intervention in neonates born at moderate altitudes.

## References

[1] Urlesberger B, Brandner A, Pocivalnik M, Koestenberger M, Morris N, Pichler G (2013). A Left-to-Right Shunt via the Ductus Arteriosus Is Associated with Increased Regional Cerebral Oxygen Saturation during Neonatal Transition. Neonatology.

[2] Ruegger C, Bucher HU, Mieth RA (2010). Pulse oximetry in the newborn: is the left hand pre- or post-ductal?. BMC Pediatrics..

[3] Bakr AF, Habib HS (2005). Normal values of pulse oximetry in newborns at high altitude. J Tropical Pediatr..

[4] Niermeyer S (2003). Cardiopulmonary transition in the high altitude infant. High Altitude Med Biol.

[5] Salyer JW (2003). Neonatal and pediatric pulse oximetry. Respiratory Care..

[6] Ewer AK, Middleton LJ, Furmston AT, Bhoyar A, Daniels JP, Thangaratinam S (2011). Pulse oximetry screening for congenital heart defects in newborn infants (PulseOx): a test accuracy study. Lancet.

[7] Arlettaz R, Bauschatz AS, Monkhoff M, Essers B, Bauersfeld U (2006). The contribution of pulse oximetry to the early detection of congenital heart disease in newborns. Eur J Pediatr.

[8] Thangaratinam S, Daniels J, Ewer AK, Zamora J, Khan KS (2007). Accuracy of pulse oximetry in screening for congenital heart disease in asymptomatic newborns: a systematic review. Arch Dis Child Fetal Neonatal Ed.

[9] Subhi R, Smith K, Duke T (2009). When should oxygen be given to children at high altitude? A systematic review to define altitude-specific hypoxaemia. Arch Dis Child.

[10] House JT, Schultetus RR, Gravenstein N (1987). Continuous neonatal evaluation in the delivery room by pulse oximetry. J Clin Monitoring.

[11] Mariani G, Dik PB, Ezquer A, Aguirre A, Esteban ML, Perez C (2007). Pre-ductal and post-ductal O2 saturation in healthy term neonates after birth. J Pediatr.

[12] Gonzales GF, Salirrosas A (2005). Arterial oxygen saturation in healthy newborns delivered at term in Cerro de Pasco (4340 m) and Lima (150 m). Reprod Biol Endocrinol..

[13] Toth B, Becker A, Seelbach-Gobel B (2002). Oxygen saturation in healthy newborn infants immediately after birth measured by pulse oximetry. Arch Gynecol Obstetr.

[14] Dimich I, Singh PP, Adell A, Hendler M, Sonnenklar N, Jhaveri M (1991). Evaluation of oxygen saturation monitoring by pulse oximetry in neonates in the delivery system. Can J Anaesthesia.

